# Peptide hydrogel with self-healing and redox-responsive properties

**DOI:** 10.1186/s40580-022-00309-7

**Published:** 2022-04-27

**Authors:** Areetha D’Souza, Liam R. Marshall, Jennifer Yoon, Alona Kulesha, Dona I. U. Edirisinghe, Siddarth Chandrasekaran, Parth Rathee, Rajeev Prabhakar, Olga V. Makhlynets

**Affiliations:** 1 Department of Chemistry, Syracuse University, 111 College Place, Syracuse, NY 13244 USA; 2 National Biomedical Center for Advanced ESR Technology, Department of Chemistry and Chemical Biology, Cornell University, Ithaca, NY 14583 USA; 3 Department of Chemistry, University of Miami, Coral Gables, FL 33146 USA

**Keywords:** Hydrogel, Self-healing and redox-responsive properties, Copper reduction, Antimicrobial wound dressing

## Abstract

We have rationally designed a peptide that assembles into a redox-responsive, antimicrobial metallohydrogel. The resulting self-healing material can be rapidly reduced by ascorbate under physiological conditions and demonstrates a remarkable 160-fold change in hydrogel stiffness upon reduction. We provide a computational model of the hydrogel, explaining why position of nitrogen in non-natural amino acid pyridyl-alanine results in drastically different gelation properties of peptides with metal ions. Given its antimicrobial and rheological properties, the newly designed hydrogel can be used for removable wound dressing application, addressing a major unmet need in clinical care.

**Supplementary Information:**

The online version contains supplementary material available at 10.1186/s40580-022-00309-7.

## Introduction

Widely accepted wet-to-dry wound dressings involve applying moist saline gauze over the wound bed to allow for moisture to evaporate and the gauze to adhere to tissue. Replacement of such dressings requires removal of the dried gauze and damages the healing wound [1–3] and is traumatic and painful for patients. Keeping the wound moist is necessary to promote healing [4], thus hydrogels that inherently retain moisture have been successfully used in wound dressings [5–9]. Antimicrobial hydrogels are especially popular because they provide both a moist environment and antimicrobial protection, resulting in improved healing outcomes [10, 11]. Moreover, hydrogels are relatively easy to replace if material could be dissolved in saline solution, yet traditional hydrogels show long dissolution times [12]. In this work, we set out to create a redox-sensitive, self-healing, antimicrobial and cytocompatible hydrogel for wound healing. While materials that possess some of these properties individually have been reported before [13–25] none of the reported materials possess all the above-mentioned properties simultaneously. Self-healing is essential for delivery of the hydrogel via a syringe, antimicrobial properties and cytocompatibility are essential for safe wound healing and redox switching offers an excellent approach to removal of the gel upon addition of a mild reductant. We started our design using a previously established antimicrobial peptide [26]. In contrast to polymer-containing hydrogels, those made of peptides are generally cytocompatible [7, 27–29]. The small size of the peptides give them an advantage over natural protein materials because modifications (such as non-natural amino acids and RGD motif) can be easily incorporated [30]. We have employed a well-established strategy to create self-healing materials via the use of metal ions to assemble hydrogels through formation of metal complexes [31–39]. We chose Cu(II) for non-covalent crosslinking because of its redox properties and ability to accelerate wound healing, including healing of diabetic ulcers and burn wounds [40–45].

## Results and discussion

### Peptide F9 with non-natural amino acid 4ʹ-pyridyl-alanine forms a hydrogel in the presence of Cu(II) ions

Recently, we developed a series of 9-residue peptides containing an unnatural amino acid for metallohydrogel preparation. We have shown that our basic design allows for semi-rational tuning of rheological properties of the resulting materials [26, 46] (Additional file 1: Table S1). We found that peptides with phenylalanine cores provide excellent stiffness and versatility in supporting metal coordination.

**Fig. 1:**
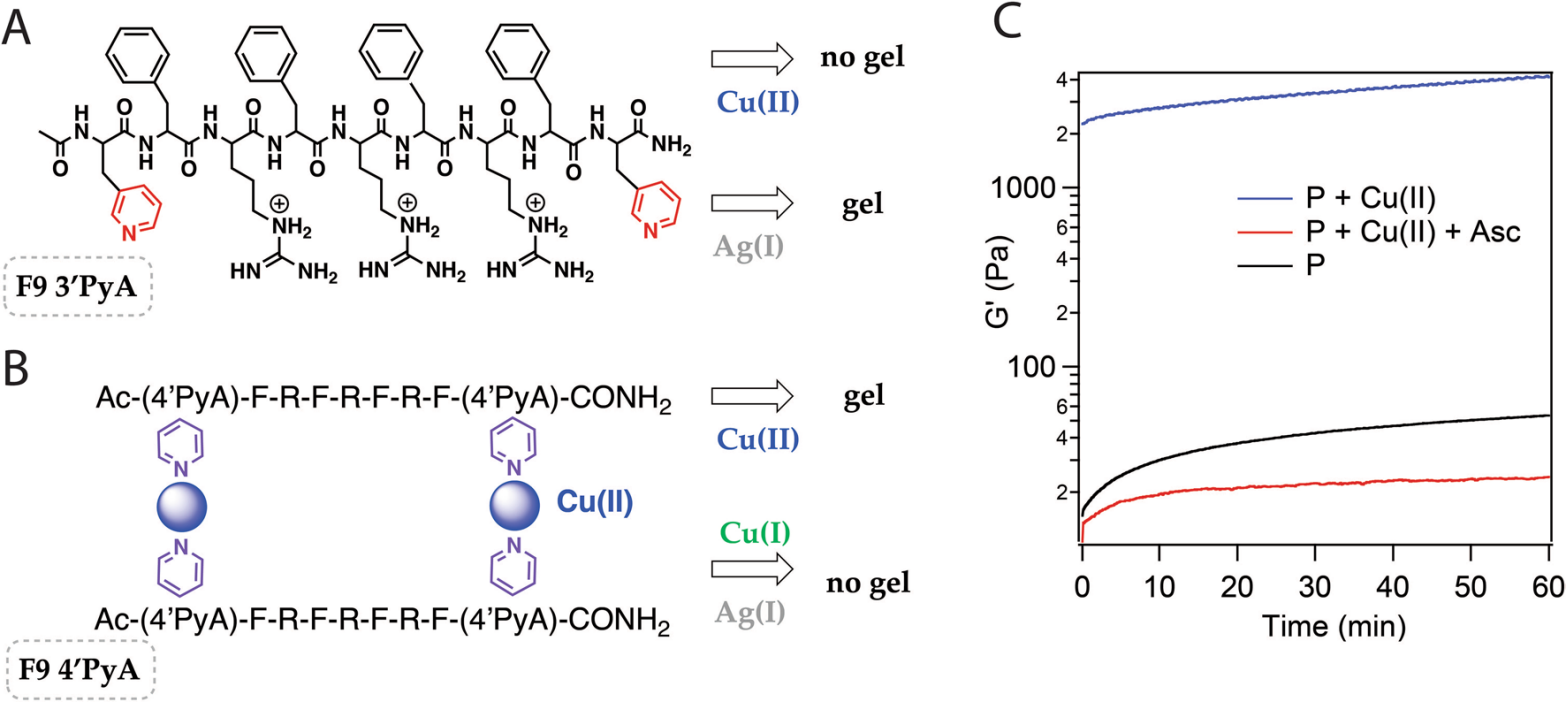
Nine-residue peptides with non-natural amino acids discussed in this work. **A** F9 3ʹPyA forms a gel with Ag(I) but not with Cu(II). **B** F9 4ʹPyA forms a gel in the presence of Cu(II) but no gel with Cu(I) or Ag(I). **C** Evolution of storage modulus for hydrogels containing 1 wt% peptide (P = F9 4ʹPyA) and 0.5 equiv Cu(II) in buffer with and without ascorbate present (5 equiv with respect to copper)

Therefore, in our present studies we focus on F9 as a core sequence. To facilitate cross-linking we have introduced pyridyl-alanine residues (3ʹPyA and 4ʹPyA) at both termini (Fig. 1). The resulting peptides, **F9 3**ʹ**PyA** and **F9 4**ʹ**PyA**, were used in subsequent studies. Cu(II) was chosen as a redox active crosslink based on its cytocompatibility (extensive utilization of copper in wound dressings [40, 47, 48] and intrauterine devices [49]) and previous success in designing redox-responsive polymers using pyridine [50], imidazole [51], 2,2ʹ-bipyridine ligand [52] and a peptide which coordinates copper ions through glutamate residues [53]. In the presence of Cu(II) **F9 4**ʹ**PyA** forms a strong gel. Location of the nitrogen in the pyridyl ring has a major impact on the structure of peptides with pyridyl-alanine side chains (Fig. 1A, B, Additional file 1: Table S1) [26]: **F9 4**ʹ**PyA** assembles into a hydrogel in the presence of Cu(II), however the same sequence with 3ʹPyA does not form a gel with Cu(II) (opposite to what we observed for Ag(I) ions). Given the similar coordination geometry preferences of Cu(I) and Ag(I) that are distinct from Cu(II), this observation supports the notion that the gelation properties are almost exclusively supported by the proper metal–ligand interactions and the peptide assemblies themselves are quite rigid as they are unable to accommodate metals in different redox states without major structural reorganization. Positioning of the metal-binding residue has a major impact on the gelation properties: placing 4ʹPyA in positions 2 and 8 of the nine amino acid peptide sequence (**F9 (2,8) 4**ʹ**PyA**) or using D-4'PyA (**F9 D-4**ʹ**PyA**) leads to reduced peptide hydrogelation (Additional file 1: Table S1). Surprisingly, despite multiple precedents that successfully utilize alternating hydrophobic residue-lysine_n_ patterns for the design of hydrogel-forming peptides, replacement of arginine residues with lysine (**FK9 4**ʹ**PyA**) is quite detrimental to gel formation (Additional file 1: Table S1). This is likely due to the short sequence length of the F9 family of peptides.

### Peptide hydrogel has antimicrobial properties with and without Cu(II)

The growth of Gram-negative bacteria, *E. coli* (ATCC 25922), was inhibited by hydrogels with and without copper ions but control samples containing only Cu(II) did not prevent bacterial growth (Additional file 1: Fig. S1A). Thus, Cu(II) ions serve a role of driving self-assembly of peptides into 3D hydrogels, enhancing their stability, but the antimicrobial properties of the hydrogel come from the peptide itself. Hydrogel samples incubated with *E. coli* cultures remain assembled and do not dissolve in the presence of bacteria, supporting the potential of the material as an antimicrobial dressing (Additional file 1: Fig. S1B).

### Cu(II)-Hydrogel assemblies are not cytotoxic

Due to the lack of cytotoxicity in similar peptide based hydrogels, as well as the extensive utilization of copper in wound dressings [40, 47, 48] and intrauterine devices [49], we anticipated excellent cytocompatibility of the copper-containing hydrogel. However, it is known that Cu(II) could participate in Fenton chemistry and produce OH radicals [54], therefore we wanted to evaluate cytotoxicity of copper and the hydrogel material. We prepared hydrogel-copper extracts and samples containing only Cu(II) by incubating with media for 72 h at 37 °C. Next, 3T3 mouse fibroblast cells (model of skin cells [55]) were treated with hydrogel extracts and their viability measured by resazurin assay. Samples prepared by soaking hydrogels (F9 4ʹPyA with Cu(II) and without Cu(II)) showed that release products from the material (peptide and copper) are not cytotoxic (Additional file 1: Fig. S2). Control samples, containing just Cu(II) ions, demonstrated that this metal is not cytotoxic for fibroblasts at the concentrations tested.

### Copper reduction significantly weakens the hydrogel

**Fig. 2:**
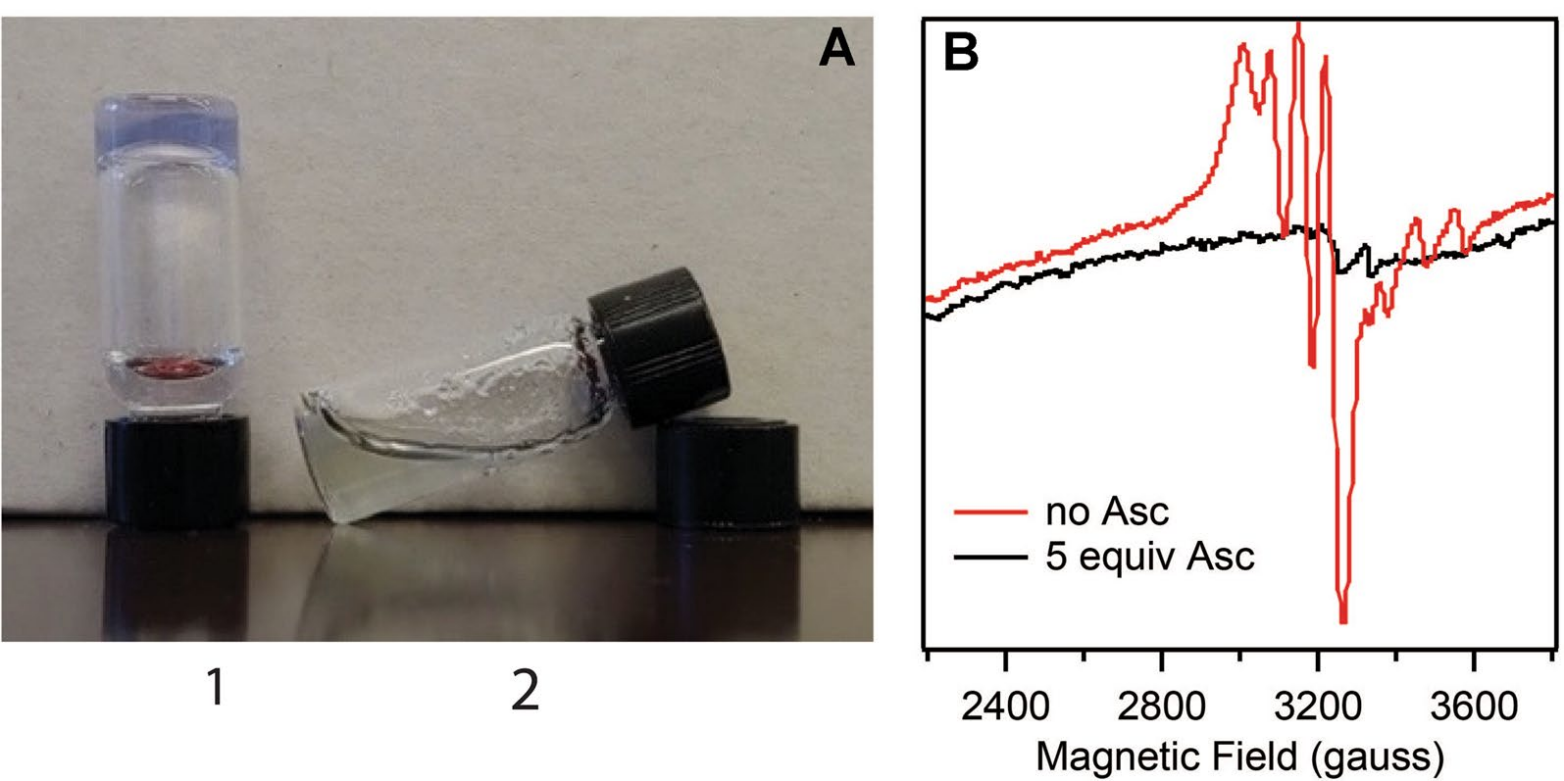
A hydrogel formed from **F9 4**ʹ**PyA** and Cu(II) dissolves after 5 min when ascorbate is added. **A** Vials containing hydrogel samples assembled using **F9 4**ʹ**PyA** (1 wt%) and 1 equiv of Cu(II) in buffer (50 mM HEPES, pH 8). Hydrogel samples (300 µL) were set by incubation at 37 °C overnight and then the buffer (50 mM HEPES, 5 mM NaCl, pH 8) containing (vial 2) or not (vial 1) ascorbate (5 equiv vs Cu(II)) was added and the picture taken in 5 min. **B** EPR spectra acquired at room temperature of the hydrogel formed by **F9 4**ʹ**PyA** and 1 equiv Cu(II). Red spectrum corresponds to 7 mM peptide/copper (1 wt% peptide) and black spectrum shows sample after reduction of Cu(II) (3.5 mM peptide/copper) to Cu(I) by ascorbate (5 equiv)

We performed an experiment where we used Cu(I) for gel formation or reduced Cu(II) to Cu(I) in the gel that had been preformed and observed much lower Gʹ values (Table 1 and Additional file 1: Table S2). In this experiment, **F9 4**ʹ**PyA** was mixed with Cu(II), buffer containing ascorbate (5 equiv) was added and then evolution of the storage modulus was measured over time. Under such conditions all copper reduced quickly to Cu(I) and remained reduced after 1 h (as confirmed by BCA assay, Additional file 1: Fig. S3), therefore Gʹ values reported in Table 1 for peptide + 0.5 equiv Cu(II) + ascorbate represent peptide with Cu(I). In addition to rheological measurements, we changed the stiffness of the hydrogel on a larger scale by reducing Cu(II) to Cu(I) and visually show that 5 min incubation with a reductant is enough to achieve dissolution of the hydrogel (Fig. 2A). Without reducing the copper crosslink, the hydrogel remained stiff even after 24 h (Additional file 1: Fig. S4). To confirm that the oxidation state of copper makes a large effect on stiffness of the hydrogel, we used Cu(I) solution prepared from CuCl and measured rheological properties for **F9 4**ʹ**PyA** with Cu(II) or Cu(I) (Additional file 1: Table S2).

**Table 1:** Rheological properties of hydrogels (1 wt% of peptides) measured at 0.5% strain, 25 °C after 1 h

Peptide name	Peptide sequence	Cu(II) equiv	Gʹ (Pa)
**F9 4**ʹ**PyA**	(**4**ʹ**PyA**)FRFRFRF(**4**ʹ**PyA**)	0	52 ± 3
		Ascorbate	104 ± 4
		0.5 + ascorbate	23 ± 3
		0.5	4113 ± 211
		1	3709 ± 770

All runs were done in triplicates, there was about 10% variation between runs. Buffer composition: 50 mM HEPES, pH 8.0. Peptide has Ac and CONH_2_ caps at N- and C-terminus, respectively

### EPR studies

Reduction of Cu(II) complexes to Cu(I) by ascorbate has been demonstrated before [52, 56, 57]; here we established the number of equivalents of ascorbate and time needed to achieve complete reduction in our copper/peptide system. We previously used EPR (electron paramagnetic resonance) to observe copper/peptide reduction by 2,6-dimethoxyphenol [58]. In this work, we used room temperature EPR to confirm that change in hydrogel stiffness is due to reduction of paramagnetic Cu(II) to diamagnetic Cu(I). Hydrogel samples were prepared using **F9 4**ʹ**PyA** (1 wt%) and substoichiometric Cu(II) (to avoid contribution of the unbound metal to the spectra). To half of the mixture, we added buffer with ascorbate to achieve hydrogel dissolution, mimicking conditions in Fig. 2A. EPR spectra in Fig. 2B show that change in stiffness is due to reduction of Cu(II) crosslink to Cu(I).

### Ratio of metal ion to peptide

**Fig. 3:**
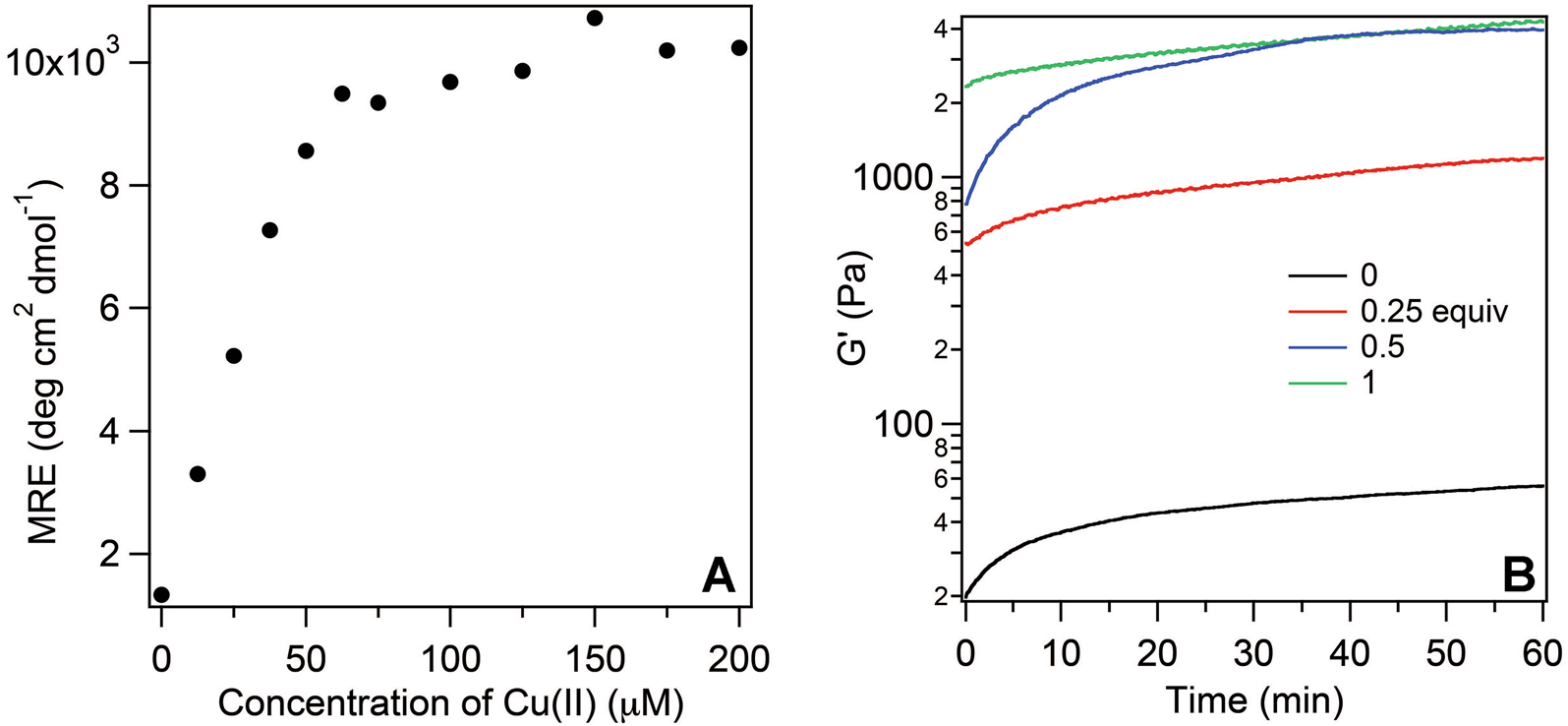
CD analysis and rheology data with variable [Cu(II)] showing that the ratio of **F9 4**ʹ**PyA** peptide and Cu(II) is 2 to 1. **A** Correlation of mean residue ellipticity (MRE) at 220 nm and copper concentration. Final concentration of peptide is 100 µM; buffer 5 mM HEPES, pH 8; pathlength of cuvettes 1 mm. Samples were prepared by incubating peptide, Cu(II) in buffer at 37 °C for 3 h. For full CD spectra see Additional file 1: Fig. S5; **B** Evolution of storage modulus over time. Gels were prepared by dissolving 2 wt% F9 4ʹPyA and Cu(II) in water and then equal volume of the buffer (75 µL; 100 mM HEPES, pH 8) was added

To establish the optimal metal:peptide ratio we measured sample ellipticity at 220 nm while keeping the peptide concentration constant and systematically varying the Cu(II) concentration. When using high concentrations of the peptide (0.5 wt%) we observed significant contribution of phenylalanine exciton coupling to circular dichroism (CD) spectra (Additional file 1: Fig. S5) [59]. To minimize this problem, we prepared samples that contained 100 µM **F9 4**ʹ**PyA** instead of 7 mM (1 wt%) concentration (Fig. 3A and Additional file 1: Fig. S6). Based on these spectra, the ratio of Cu(II):peptide is 1:2, which is consistent with four 4ʹPyA ligands around each Cu(II) center, presumably with water ligands completing a distorted octahedral coordination geometry. It is possible that the ratio of peptide:copper is different when a higher concentration of peptide is used. To avoid contribution of exciton coupling to the spectra, we prepared hydrogel samples using peptides with a leucine core instead of phenylalanine (Additional file 1: Fig. S7). Leucine peptide L9 4ʹPyA forms a gel in the presence of Cu(II) as shown in Additional file 1: Table S1 and is a good substitute for CD studies. Using L9 4ʹPyA we also observed that 0.5 equiv of Cu(II) is enough to form a hydrogel and higher concentrations of Cu(II) do not yield higher CD signal. This result is also consistent with Cu(II):peptide ratio of 1:2. We further confirmed the ratio of Cu(II):peptide as 1:2 using rheological studies (Fig. 3B).

### The gel formed by F9 4ʹPyA and copper is self-healing

**Fig. 4:**
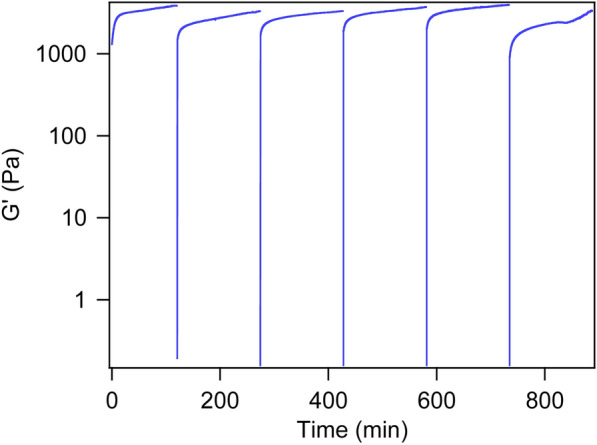
Shear recovery of the hydrogel prepared from **F9 4**ʹ**PyA** (1 wt%) with Cu(II) (1 equiv) in buffer (50 mM HEPES, pH 8). Hydrogel was subjected to 1000% strain at 6.283 rad/s for 30 s, 25 °C, followed by an oscillation time sweep experiment (0.5% strain) for 2 h to check the sample’s recovery after shear. The numbers for the hydrogel samples reported for 2-h recovery are in Additional file 1: Table S3

Given the hydrogel lacks covalent crosslinks, it can recover its storage modulus after applying strain; hydrogels with such characteristics are self-healing and can be delivered using a syringe, offering very desirable therapeutic applications [34, 60–62]. In this work, we demonstrate that developed hydrogel with Cu(II) crosslinks is self-healing. For shear recovery test, the hydrogel sample formed by mixing **F9 4**ʹ**PyA** (1 wt%) and Cu(II) (1 equiv) was subjected to strain for 30 s followed by an oscillation time sweep experiment for 2 h to check the sample recovery. We observed at least 5 cycles of recovery for the hydrogel (Fig. 4 and Additional file 1: Table S3). Hydrogels assembled from **F9 4**ʹ**PyA** (1 wt%) and Cu(II) (0.5 equiv) also demonstrated self-healing behaviour (Additional file 1: Fig. S8).

### Peptide assemblies with Cu(II) have a β-sheet secondary structure and form fibrils

**Fig. 5:**
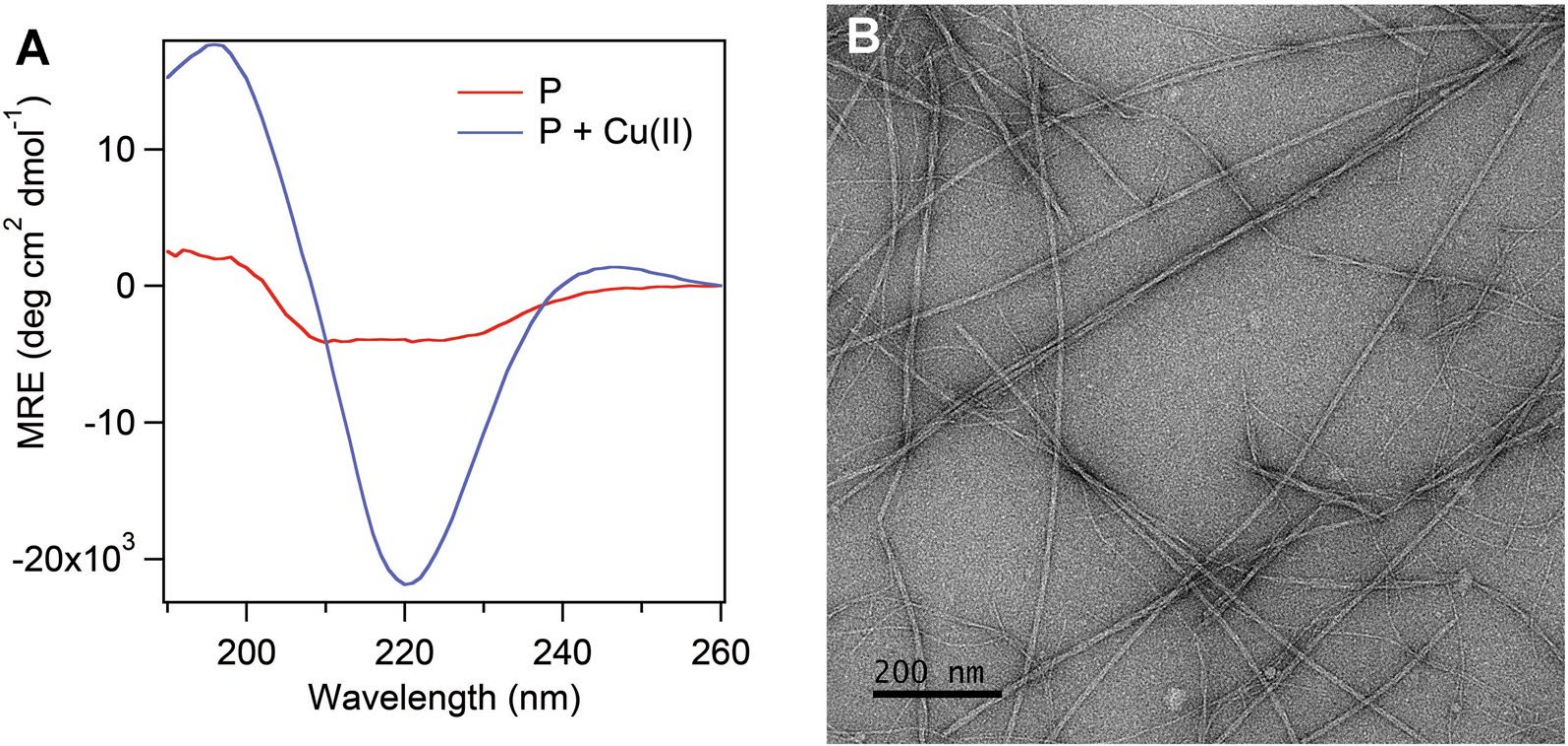
Peptide assembles into β-sheets in the presence of Cu(II) and forms fibrils. **A** CD spectra of **L9 4**ʹ**PyA** peptide with 1 equiv of Cu(II). Hydrogel samples were assembled from **L9 4**ʹ**PyA** (1 wt%, 7.5 mM) with (P + Cu(II)) or without Cu(II) (P) in buffer (50 mM HEPES, pH 8), incubated overnight at 37 °C and the spectra were measured using 0.1 mm cuvette. **B** TEM image showing the fibrils of the hydrogel formed from **F9 4ʹPyA** (1 wt%) and Cu(II) (1 equiv) in buffer

To probe the secondary structure of the peptide we performed a CD study (Fig. 5A). Because of exciton coupling due to Phe residues in the sequence (Additional file 1: Fig. S5), we used **L9 4**ʹ**PyA** instead of **F9 4**ʹ**PyA**. **L9 4**ʹ**PyA** in the presence of Cu(II) showed a CD signature characteristic of β-sheets. Transmission electron microscopy (TEM) images of **F9 4**ʹ**PyA** with Cu(II) (Fig. 5B) show the formation of fibril-like structures as expected.

### Computational modeling

**Fig. 6:**
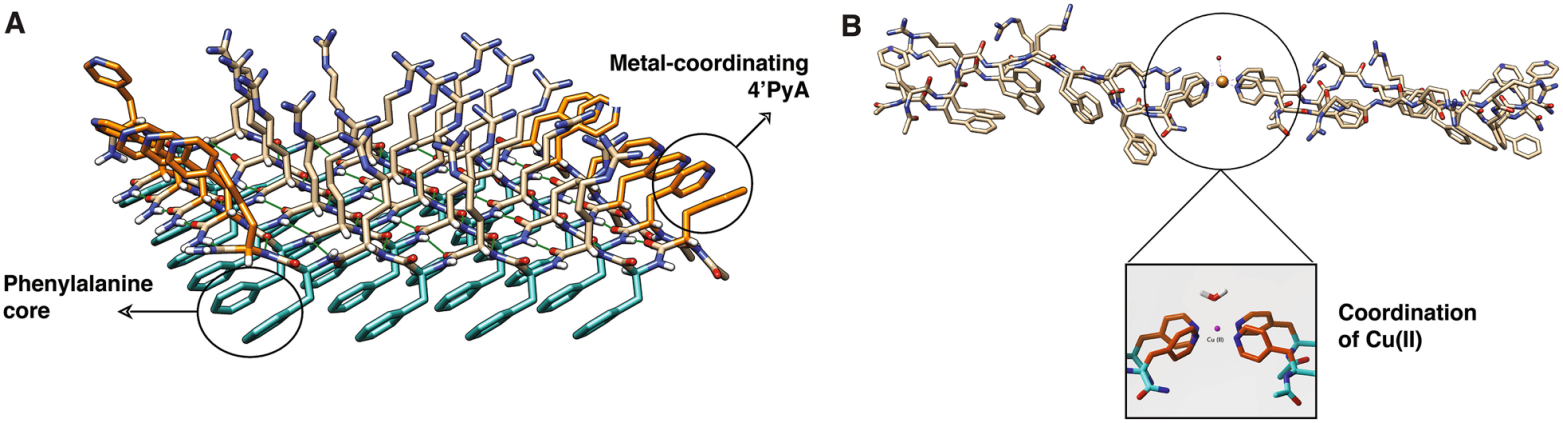
Computational model of the peptide-based hydrogel without (**A**) and with (**B**) copper(II) ion

Many metallohydrogels with various sequences have been reported, yet structural studies of these assemblies remain very scarce [63]. To fully advance our understanding of metallohydrogels we must understand the structural basis for this function. We used experimental data to build a high-quality structure for computational modeling. The equilibrated structure of the metal-free hydrogel derived from 200 ns all-atom MD simulations is shown in Fig. 6A. It is a two-layer sandwich like structure (Additional file 1: Fig. S9) stabilized by non-covalent interactions such as hydrogen bonding and π–π stacking. For instance, the top and bottom layers connected by π–π stacking between the Phe residues of the peptide. This structure is further stabilized by the strong hydrogen bonding between the amide and carbonyl groups of the two adjacent strands. Additionally, all positively charged Arg residues of these peptides are oriented upwards perpendicular to the plane of the hydrogel. They interact with solvent water molecules through hydrogen bonding. Furthermore, the terminal 4ʹPyA groups interact with each other through π–π stacking. This structure contains 96.3% $\beta$ sheet character. The interactions of the Cu(II) ion with the 4ʹPyA residues of this structure were investigated using more accurate hybrid quantum mechanics/molecular mechanics (QM/MM) optimizations. In the optimized structure, two pyridyl alanine residues from different peptides coordinate to the metal ion at the equatorial positions, while the solvent water molecule occupies the axial position (Fig. 6B). This complex exists in a square-pyramidal conformation. This specific binding mode could be the reason that the peptide with 3ʹPyA ligands are unable to form hydrogels in the presence of the Cu(II) ion. The pyridyl rings of these residues are sterically hindered to orient at the equatorial position needed for the metal binding.

## Conclusions

We have rationally designed a short, nine-residue peptide **F9 4**ʹ**PyA** that assembles into a redox-responsive, antimicrobial metallohydrogel upon addition of Cu(II). The resulting self-healing material can be rapidly reduced by ascorbate under physiological conditions and demonstrates a remarkable 160-fold change in hydrogel stiffness upon reduction. Cu(II)- F9 4ʹPyA gel undergoes shear-thinning under strain with complete gelation recovery once the strain has been removed. While hydrogels with various properties have been reported before, this work, to our knowledge, provides the first example of the material that combines antimicrobial, redox-active and self-healing properties. The computational modeling provided information regarding the coordination mode of Cu(II) and the ligands in the hydrogel, and will help to guide future designs of hydrogels. Nine-residue peptides are simple and inexpensive to produce, opening the path to the large-scale production of these materials. Given its antimicrobial and rheological properties, the newly designed hydrogel can be used for removable wound dressing application, addressing a major unmet need in clinical care.

## Supplementary Information

**Additional file 1. Contains supplementary figures S1–S17, tables S1–S3 and “Materials and Methods” section.**

## Data Availability

The datasets used and/or analysed during the current study are available from the corresponding author on reasonable request.

## Abbreviations

PyA: Pyridyl-alanine
CD: Circular dichroism
EPR: Electron paramagnetic resonance
