# Supplementary material for: Peptide hydrogel with self-healing and redox-responsive properties
Source: Nano Converg. 2022 Apr 27;9:18. doi: 10.1186/s40580-022-00309-7 (PMC9046503; doi:10.1186/s40580-022-00309-7)
Supplement: Supplementary file 1 — Additional file 1. Contains supplementary figures S1–S17, tables S1–S3 and “Materials and Methods” section. [file 40580_2022_309_MOESM1_ESM.pdf]

MATERIALS AND METHODS

Peptide synthesis and purification. The peptides were synthesized by manual Fmoc solid-phase synthesis at 70°C using Rink amide resin (ChemImpex) and Fmoc-protected amino acids (GL Biochem Shanghai Ltd.) in accordance with previously reported protocols.(1) Fmoc-3-(4′-pyridyl)-L-alanine couplings were performed at room temperature for 1 hour and arginine residues were coupled for 10 minutes at 70°C. Peptides were acetylated at N-terminus at room temperature for 20 minutes. Cleavage of the peptides from the resin and side-chain deprotection were simultaneously achieved by treatment with a mixture of trifluoroacetic acid (TFA)/H_2O/triisopropyl silane (TIS) (95:2.5:2.5, vol/vol) for 2 hours at room temperature. The crude peptides were precipitated and washed with cold methyl-tert-butyl ether and purified on a Varian ProStar 210 preparative reverse phase High Performance Liquid Chromatography system with a C4 preparative column (Vydac), using a linear gradient of solvent A (0.1% TFA in MilliQ water) and solvent B (90% CH_3CN, 9.9% MilliQ water, 0.1% TFA). A gradient of 35-65% of solvent B was used at a flow rate of 15 ml/min for 30 minutes to purify the peptides. The identities of the peptides were confirmed using either a Shimadzu LCMS-8040 (H_2O/MeCN/TFA = 40/60/0.1 at a flow rate of 0.2 mL/min) or a Bruker Autoflex III Smartbeam MALDI-TOF mass spectrometer (CHCA matrix). Purity of the obtained peptides was evaluated on an Agilent Infinity II 1260 with an analytical Zorbax Eclipse XDB-C18 column (4.6 mm × 150 mm). Peptide stock solutions were prepared from the lyophilized powder (<90% purity). Analysis of purified peptides is shown in Figures S10-S17.

Peptide stock preparation. Pure lyophilized F9 4′PyA peptide was dissolved in cold MilliQ water to prepare a 14 mM stock solution (2 wt%). L9 4′PyA and FK 4′PyA peptide were dissolved in MilliQ water to prepare a 15 mM stock solution (2 wt%). The peptide solution was then spin-filtered (0.2 μm PES filter) and centrifuged at 6,500 rpm for 10 minutes. The concentrations of the peptide were determined by measuring absorbance at 255 nm on an Agilent 8453 UV-Vis spectrophotometer using ε_{255}= 4439 M^{-1} cm^{-1}. The peptide stocks were then aliquoted into 75 μL in each microcentrifuge tube and lyophilized. The lyophilized aliquots were stored at -20°C until further use.

Circular dichroism spectroscopy. The CD spectra were acquired on the Jasco J-715 CD spectrometer collecting ten scans (4 s averaging time) for each spectrum and using a quartz cuvette. The measurements were performed on samples containing 0.5 wt% peptide in buffer (50 mM HEPES, pH 8) in the presence and in the absence of $CuSO_4$. The hydrogel samples were incubated at 37°C overnight to ensure gel formation before measurements. Care was taken that the sample absorbance never exceeded 1.5 at all wavelengths to produce reliable ellipticity values. Measurements were taken in cuvette with 0.1 mm path length. To determine the stoichiometry of F9 4′PyA to Cu(II) at a lower concentration, peptide (100 μM) was mixed with different concentrations of Cu(II) (12.5, 25, 37.5, 50, 62.5, 75, 100, 125, 150, 175 and 200 μM of $CuSO_4$) in buffer (5 mM HEPES, pH 8). The peptide/Cu(II) samples were aged at 37°C for three hours before recording measurements. The measurements were acquired using 1 nm bandwidth, collecting ten scans (4 s averaging time) for each spectrum and using a quartz cuvette of 1 mm pathlength.

Mean residue ellipticity (MRE, $deg*cm^{2}*dmol^{-1}$) values were calculated using the following equation, where θ is ellipticity (mdeg), l is pathlength (cm), C is peptide concentration (M), N is number of residues.

$$MRE = \theta/(10*C*l*N)$$

Peptide Hydrogel Rheometer Procedure. The mechanical properties of the peptide hydrogel (the storage (G′) and loss (G′′) moduli) were measured on an ARG2 rheometer (TA Instruments). An aliquot of powdered peptide was dissolved in ice-cold water (75 μL) to prepare a 2 wt% (14 mM for F9 4′PyA) stock and mixed with a vortex mixer (GeneMate, BioExpress) for 10 s. The peptide solution was centrifuged (Eppendorf, Microcentrifuge 5424) for 5 min at 6,000 rpm to remove bubbles. Ice-cold buffer (100 mM HEPES, pH 8) was added into the peptide solution and gently pipette mixed (1:1 ratio of peptide and buffer solutions). Sample solution (75 μL) was pipetted onto the rheometer equipped with a solvent trap and the moduli measured on a cone and plate geometry with a 2° 20 mm cone at a gap of 57 μm. A solvent trap accessory was used to prevent solvent evaporation. Gelation was monitored by performing oscillation time sweep experiments at 25°C with a constant 0.5% strain and 6.283 rad/s angular velocity for 1 hour. For the sample with ascorbate, we mixed peptide and Cu(II) in water and then added degassed buffer containing sodium ascorbate (concentration in the final sample: 7 mM F9 4′PyA, 3.5 mM Cu(II), 17.5 mM ascorbate). After gentle mixing of the sample, evolution of the storage modulus was recorded. We show that mixing time (about 2 min) is sufficient for copper reduction and copper remains reduced for at least 1 hr as measured by bicinchoninic acid (BCA) assay. In shear recovery tests, samples are subjected to 1000% strain at 6.283 rad/s for 30 s, followed by an oscillation time sweep experiment (0.5% strain) for 2 hours to check the sample's recovery after shear. Experiments are completed in triplicate to ensure reproducibility.

Transmission electron microscopy. Hydrogel samples, containing F9 4′PyA peptide in the presence of 1 equivalent of $CuSO_4$ in buffer (50 mM HEPES, pH 8) were prepared and incubated at 37°C overnight to ensure gel formation. After preparation, 3 μL aliquots were applied to carbon-coated 200-mesh copper grids (Ted Pella) and left for 2 minutes. Excess liquid was wicked away with filter paper, and grids were then stained with one 5 μL drop of 2% (w/v) uranyl acetate, followed by immediate blotting. A second 5 μL drop of 2% (w/v) uranyl acetate was applied and left on the grid for 20 seconds. This was then blotted away, and grids were stored under vacuum until imaging. Samples were viewed with a JEOL-JEM 2100F Field Emission electron microscope at an acceleration voltage of 200 kV. Electron micrographs were recorded on a Gatan OneView 4K CCD camera.

Inhibition of bacterial growth assay. $CuSO_4$ was dissolved in MilliQ water to make a 50 mM stock solution. MilliQ water, buffer (100 mM HEPES, pH 8) and $CuSO_4$ solution were sterilized by filtering through 0.22 μm sterile centrifugal filters. The lyophilized peptide aliquot was dissolved in sterile MilliQ water to make a 14 mM solution (2 wt%) of F9 4′PyA. The hydrogel sample with Cu(II) was prepared by dissolving peptide stock in water and then Cu(II) was added (1 equiv of Cu(II), total volume of peptide+Cu(II) in water was 300 μL). Then the mixture was aliquoted into wells of a 48 well plate (Becton, Dickinson and Company) in triplicates (100 μL in

each well) and the buffer was added (100 μL). For the samples of peptide hydrogel without Cu(II), water was added to dissolve the lyophilized peptide, followed by buffer. The plate was covered with a Breathe-Easy permeable membrane (WorldWide Medical Products, Inc) and incubated at 37°C overnight to ensure gel formation. Control samples without the peptide were prepared by mixing water and buffer (or water with Cu(II) and buffer) instead of peptide solution and buffer. The culture of *E. coli* (ATCC 25922) was started by mixing overnight culture (100 μL) in LB (5 mL) and the resulting culture was grown at 37°C, shaking at 150 rpm for about 2 hours until OD_{600} = 0.3. The culture was then diluted to OD_{600} = 0.001 with sterile media and this diluted culture was added to each well (300 μL). 'Media' sample, was prepared by mixing water with Cu(II) (100 μL) and buffer (100 μL) and then sterile LB medium was added (300 μL). The plate was then covered with a new membrane and placed in a shaker at 37°C for 24 hours (150 rpm). Next day, 200 μL of the culture was carefully removed from the top and transferred to a 96 well plate (Cellstar from Greiner Bio-One) to measure OD_{600} using a plate reader (Multiskan Spectrum from Thermo Labsystems).

To evaluate the stability of hydrogel material with *E. coli* culture, we followed the below protocol. A solution of F9 4'PyA peptide in sterile cold water (2 wt% for the peptide) was measured into Eppendorf tubes (100 μL in each). Then ice-cold sterile buffer (100 μL of 100 mM HEPES, pH 8) was added to each tube. Preparation of hydrogels containing peptide-copper: peptide was dissolved in sterile cold water (2 wt% for the peptide), then sterile ice-cold $CuSO_4$ (50 mM) was added to account for 1 equiv of Cu(II) vs peptide, this solution was placed into Eppendorf tubes (100 μL in each), ice-cold sterile buffer (100 μL of 100 mM HEPES, pH 8) was added to each tube. Reagents were sterilized using filtration through 0.22 μm PES syringe filters (Santa Cruz Biotechnology. Inc). The tubes were punctured using a sterile needle, covered with a Breath-Easy permeable membrane, and incubated at 37°C overnight. The culture of *E.coli* was prepared by adding *E.coli* glycerol stock (ATCC 25922, 5 μL) to LB medium (5 mL) and grown overnight shaking at 150 rpm at 37°C. Next day this overnight culture (100 μL) was taken and added to fresh LB medium (5 mL) and the culture was grown until the OD_{600} reached a value around 0.3. The culture was diluted to OD_{600} = 0.001 and this diluted culture (300 μL) was introduced to the gels. The samples in the tubes were covered again with a Breath-Easy permeable membrane and shaken at 150 rpm at 37°C for 24 hr. To visualize the hydrogel, the culture on top was removed and then DMEM medium (300 μL) was added.

Cytocompatibility experiment. The F9 4'PyA (1 wt %) without copper was prepared by dissolving three lyophilized peptide aliquots in sterile MilliQ water (ice-cold, 75 μL to each tube). Peptide solutions in water were combined and 50 μL of this solution was pipette mixed with an equal volume of buffer (50 μL of 100 mM HEPES, pH 8). The final concentration of F9 4'PyA was 7 mM (1 wt%) in buffer (50 mM HEPES, pH 8). $CuSO_4$ was dissolved in MilliQ water to prepare a 50 mM stock solution and syringe-filtered by passing through 0.22 μm sterile centrifugal filter (PES filter, Santa Cruz). For the F9 4'PyA (1 wt %) peptide containing copper (0.5 equiv), the samples were prepared by dissolving three lyophilized peptide aliquots in ice-cold sterile MilliQ water (64.5 μL for each aliquot) and then $CuSO_4$ solution (ice-cold, 10.5 μL of 50 mM) was added to each tube. These peptide-copper stocks were combined and 50 μL of the solution was pipette

mixed with an equal volume of buffer (50 μL of 100 mM HEPES, pH 8). The final concentration of F9 4'PyA was 7 mM and Cu(II) was 3.5 mM in buffer (50 mM HEPES, pH 8.0). The F9 4'PyA (1 wt %) containing copper (1 equiv) was prepared by dissolving three lyophilized peptide aliquots in ice-cold MilliQ water (54 μL for each aliquot) and then $CuSO_4$ solution (ice-cold, 21 μL of 50 mM) was added to each tube. These peptide-copper stocks were combined and 50 μL of the solution was pipette mixed with an equal volume of buffer (50 μL of 100 mM HEPES, pH 8). The final concentration of both F9 4'PyA and Cu(II) was 7 mM in buffer (50 mM HEPES, pH 8). All samples were prepared in triplicate and incubated at 37°C overnight to ensure gel formation. The control samples with just Cu(II) were prepared by adding water instead of the peptide stock. These control samples were prepared before cytotoxicity experiment as no extract needed to be obtained here. After hydrogel formation, 700 μL of DMEM media (Corning) with 10% fetal bovine serum (FBS, Gibco) was added to each tube, and the samples were incubated for 72 hr at 37 °C without shaking. NIH/3T3 (ATCC® CRL-1658™) cells were obtained from Monroe lab (SBI) as a frozen aliquot in DMSO (0.5 million cells per vial at 20th passage). The cells were propagated in DMEM with L-glutamine, 4.5g/L glucose, and sodium pyruvate (Corning) supplemented with 10% FBS and 1% penicillin-streptomycin mix (Gibco). After each trypsinization, cells were plated in T75 flask and grown until 80% confluency at 37°C, 5% CO_2 and high humidity. After trypsinization, cells were counted, diluted in the supplemented DMEM and dispensed into 96-well plate in 100 μL aliquots approximately 24 hr before testing at concentrations of 10,000 cells/well. After the extracts (100 μL) were added to wells (n = 3) for each tested group, the cells were incubated for 3 hr under the cell culture conditions. Cells without extract treatment (fresh cell culture media was added instead of extracts) were used as a positive control and cells treated with 3% H_2O_2 (Fisher) were used as a negative control. A Resazurin assay was performed to evaluate cell viability. Resazurin (Sigma) was added to the cells to the final concentration of 0.67 mM and fluorescence was measured after 4 hours of incubation using Biotek Synergy 2 plate reader (filters Ex 550/25, Em 590/35 nm). Percent cytocompatibility was calculated as $(F_{extract} - F_{negative\ control})/(F_{positive\ control} - F_{negative\ control}) \times 100$ for an average of 3 runs.

EPR experiments. An aliquot of lyophilized 2 wt% F9 4'PyA peptide (75 μL of 14 mM solution) was dissolved in ice-cold MilliQ water (54 μL) and then $CuSO_4$ solution (ice-cold, 21 μL of 50 mM) was added. The peptide/Cu(II) solution was centrifuged for 5 min at 6,500 rpm to remove bubbles. Peptide/Cu(II) solution (75 μL) was gently pipette mixed with an equal volume of buffer (75 μL, 100 mM HEPES, pH 8). The final concentration of F9 4'PyA is 7 mM containing 7 mM of Cu(II) in 50 mM HEPES pH 8. The hydrogel sample was kept at 37°C overnight. A portion of this sample (F9 4'PyA/Cu(II), 25 μL) was loaded into microcapillary pipet (Kimble Chase #71900-50) using a Hamilton syringe, spun down and then EPR measurements were carried out within 24 hours. After overnight incubation, the second portion of the F9 4'PyA/Cu(II) hydrogel sample (75 μL), was mixed with degassed buffer (75 μL, 50 mM HEPES, 5 mM NaCl, 35 mM ascorbate, pH 8) and the tube was inverted several times and incubated for about 15 mins. The reduced sample was then transferred to the microcapillary tube. The samples were sealed after transferring to the tubes and the spectra collected at Cornell University the following day. EPR experiments were carried out on a Bruker E500 spectrometer with the super hi-Q resonator operating at ~ 9.32 GHz (X-Band), room temperature and the following experimental parameters: modulation frequency,

100 kHz; modulation amplitude, 4G; and microwave power, 6.325 mW; numbers of scans, 4-9. Data acquisition and manipulation were carried out using the Xepr software.

Solubilization of hydrogel. Hydrogel samples (300 μL) containing 1 wt% (7 mM) F9 4′PyA with 1 equivalent of Cu(II) in buffer (50 mM HEPES, pH 8) were formed in two glass vials and kept overnight at 37°C. To reduce Cu(II) in the hydrogel, degassed buffer (300 μL) containing 5 equivalents of ascorbate (35 mM) in buffer (50 mM HEPES, 5 mM NaCl ,pH 8) was added to one of the vials and swirled about 10 times. Buffer without ascorbate (300 μL) was added to the other vial for a comparison. The vials were inverted to check if the solution was in hydrogel or solution form. Degassed buffer was prepared on a Schlenk line using nitrogen gas and then the buffer was stored inside glove box and aliquots taken out before the experiment.

BCA assay. The rate and length of Cu(II) reduction was measured using a BCA assay (BCA = bicinchoninic acid). Both Cu(II) in buffer and Cu(II) and peptide at hydrogel concentration were investigated using this method. For Cu(II) samples, samples (3.5 mM copper) were prepared by mixing 50 mM stock of Cu(II) in water (10.5 μL), MilliQ water (64.5 μL) and degassed buffer (75 μL of 100 mM HEPES, pH 8) containing 5 equiv ascorbate (35 mM ascorbate). Then the aliquots (20 μL) of the sample were taken for each time point and dissolved in MOPS (25 mM, pH 6), and BCA (250 uM final concentration). The absorbance at 563 nm was measured and amount of Cu(I) present is estimated using extinction coefficient ε_{563}= 7,900 M^{-1} cm^{-1}. For the sample containing both Cu(II) and the peptide at hydrogel concentration (150 μL total volume), a mixture containing peptide (7 mM), Cu(II) (3.5 mM) and degassed buffer (50 mM HEPES, pH 8) and 5 equiv ascorbate (17.5 mM ascorbate) was used; 20 μL aliquots from the reduced hydrogel sample were taken for each time point and dissolved in MOPS buffer with BCA and the concentration of reduced $Cu(I)BCA_2$ was measured as described above.

Molecular dynamics (MD) simulations. An initial 6 x 2 F9 4′PyA bilayer peptide aggregate was built from the solid-state NMR structure of a similar short peptide (PDB ID - 5ugk) (2) using the YASARA program (3). The Amber force field parameters of the unnatural amino acid 4′ PyA were developed utilizing the Antechamber tool in the Amber software package (4). Due to the symmetric nature of the FRFRFRF sequence, only a parallel conformational was used. The all-atom 200 ns Molecular Dynamic (MD) simulations were performed using the AMBER99SB-ILDN forcefield and TIP3P water model as implemented in the GROMACS program (5). The structure was then placed at the center of the box with 6 x 6 x 6 nm^3 dimension and periodic boundary conditions (PBC) were employed. The bond constraints were imposed using the LINCS algorithm (6) and inter atomic electrostatic interactions were treated using the particle mesh Ewald method (PME) (7). The velocity-rescale thermostat and Berendsen pressure coupling models were utilized for the required pressure/temperature couplings during the relaxation (8, 9). The total charge of the system is +36 is due to the presence of multiple positively charged Arg residues. This system was neutralized using a suitable number of randomly placed Na^+ and Cl^- ions.

QM/MM optimizations. The Cu(II) binding to the model of hydrogel was studied by optimizing a bi-peptide (2 x 2 monomers) taken from the previous MD equilibrated structure using the two-

layer QM/MM ONIOM method (10). All QM/MM optimizations were performed using the Gaussian 09 package (11). In the QM/MM models, Cu(II) ions, 4′ PyA and a water molecule were included in the high-level QM layer, while the rest of the system in the low-level MM layer. All atoms, except Cu, in the QM layer were treated at the B3LYP/6-31G* level (12, 13), while atoms in the MM layer using the Amber99 force field (4). The Cu atom was treated with the Lanl2dz basis set with a suitable Hay-Wadt effective core potential (14).

Table S1. Rheological properties of hydrogels (1 wt% of peptides) measured at 0.5% strain, 25°C after 1 hr. All runs were done in triplicates, variation between runs was approximately 10%. Buffer composition: 50 mM HEPES, pH 8.0. All peptides have Ac and $CONH_2$ caps at N- and C-terminus, respectively.

Peptide name	Peptide sequence	Ag(I) equiv	Cu(II) equiv	G′ (Pa)
L9 3′PyA	(**3′PyA**)LRLRLRL(**3′PyA**)	0	0	67 ± 1
		1	0	1014 ± 23
		0	1	154 ± 11
L9 4′PyA	(**4′PyA**)LRLRLRL(**4′PyA**)	0	0	27 ± 1
		1	0	25 ± 1
		0	0.5	775 ± 2
		0	1	1461 ± 226
F9 2′PyA	(**2′PyA**)FRFRFRF(**2′PyA**)	0	0	20 ± 1
		0	1	5 ± 2
F9 3′PyA	(**3′PyA**)FRFRFRF(**3′PyA**)	0	0	65 ± 6
		1	0	22690 ± 528
		0	1	69 ± 2
F9 4′PyA	(**4′PyA**)FRFRFRF(**4′PyA**)	0	0	52 ± 3
		0	0.5 + ascorbate	23 ± 3
		0	ascorbate	104 ± 4
		0	0.5	4113 ± 211
		0	1	3709 ± 770
FK9 4′PyA	(**4′PyA**)FKFKFKF(**4′PyA**)	0	0	123 ± 3
		0	0.5	2613 ± 37
F9 (2,8) 4′PyA	F(**4'PyA**)FRFRF(**4'PyA**)F	0	0	62 ± 5
		0	0.5	123 ± 3
F9 D-4′PyA	(**4′^{D}PyA**)FRFRFRF(**4′^{D}PyA**)	0	0	53 ± 3
		0	0.5	2995 ± 84

A B

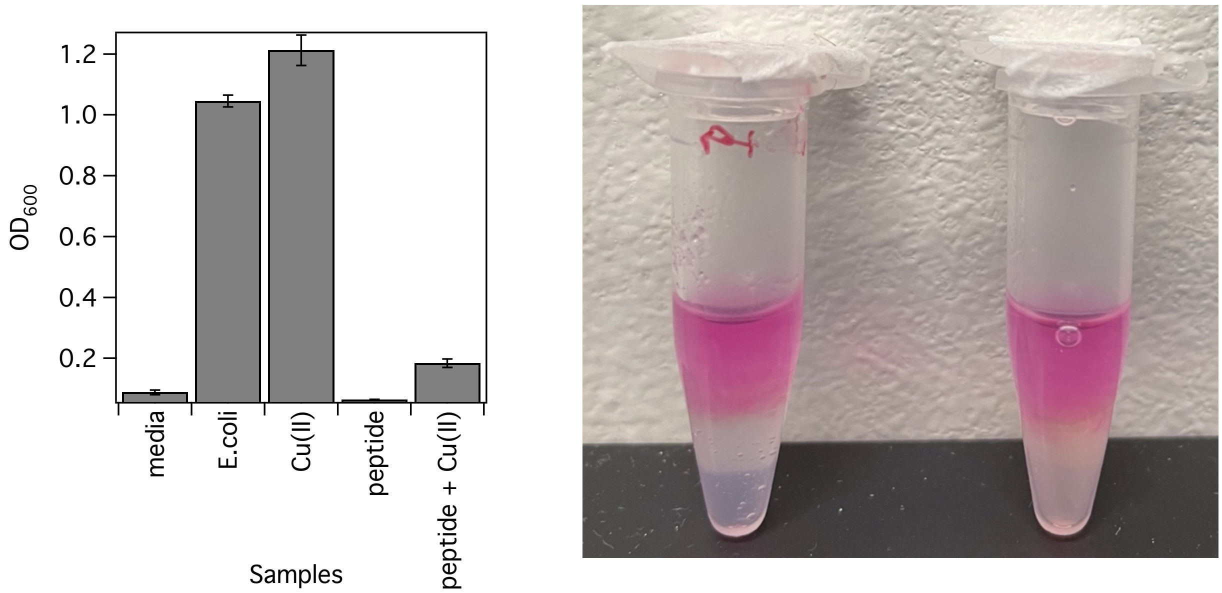

Figure S1. A) Inhibition of bacterial growth. Hydrogel samples were formed from peptide F9 4′PyA (1 wt%) in both the presence and absence of copper (1 equiv of Cu(II) with respect to the peptide). All samples excluding the negative control (media) contained *E. coli* culture (A). B) Hydrogels assembled in the presence (left) and absence (right) of Cu(II) remain intact after 24 hr incubation with *E. coli* culture. DMEM medium was added to the hydrogel for better contrast.

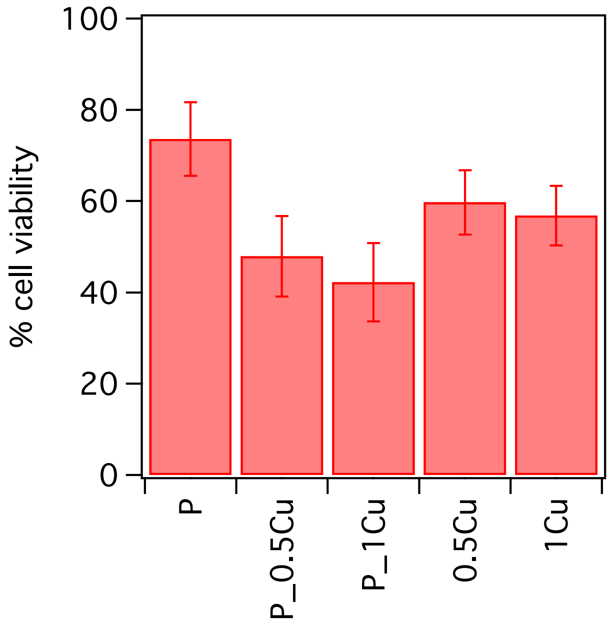

Figure S2. Hydrogel extracts containing F9 4'PyA and Cu(II) or extracts containing just Cu(II) are cytocompatible with 3T3 mouse fibroblast cells as measured by resazurin assay. Samples 0.5Cu and 1Cu contained only Cu(II) ions and no peptide; samples P_1Cu and P_0.5Cu correspond to extracts prepared by soaking peptide-copper hydrogels in media for 72 hr; sample P corresponds to extract prepared from peptide hydrogel (no copper). Control samples with just copper demonstrate that treatment of fibroblast cells with this ion does not result in significant cell death. Data shown are the averages of 3 trials with standard deviations as error bars.

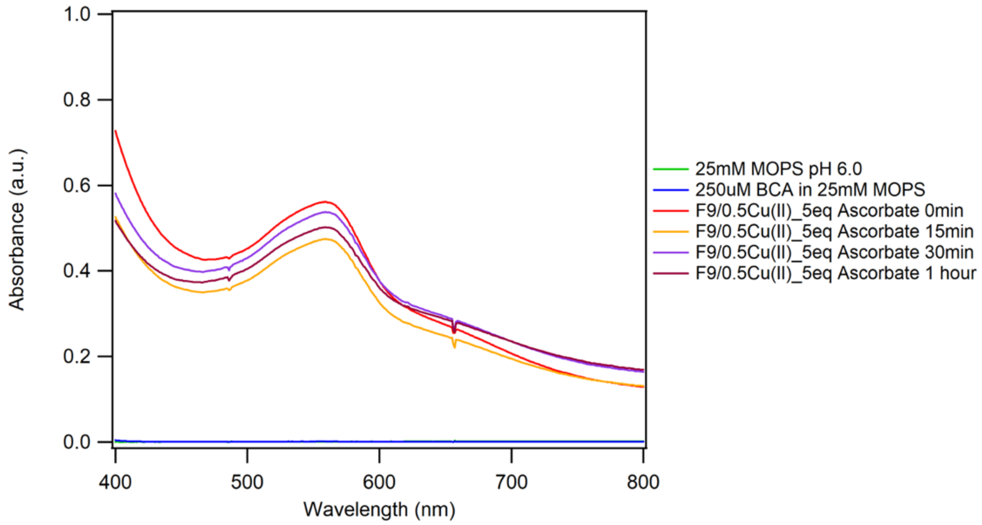

Figure S3. Absorbance of Cu(I)BCA_2 for the sample containing peptide, Cu(II) and ascorbate, which was diluted into MOPS buffer (25 mM MOPS, pH 6), and BCA (250 μM final concentration). Sample was prepared by mixing Cu(II) (3.5 mM), peptide F9 4′PyA (7 mM) and ascorbate (17.5 mM). At various timepoints aliquots were taken (20 μL) and diluted 50-fold into MOPS buffer with BCA. The absorbance at 563 nm was measured and amount of Cu(I) present is estimated using extinction coefficient of Cu(I)BCA_2 ε_{563}= 7,900 M^{-1} cm^{-1}. We calculated a concentration of 55 μM copper instead of 70 μM, which would correspond to all the copper reduced (even for the samples that contained just 3.5 mM Cu(II) in buffer). This mismatch is probably due to less-than-optimal pH of the BCA samples (atomic absorption standard containing some nitric acid showed 70 μM copper). We assumed that all copper was reduced for at least one hour because absorbance remained largely unchanged after this time.

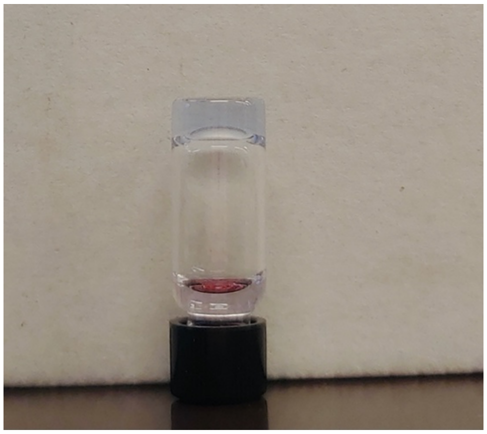

Figure S4. A hydrogel formed from F9 4′PyA and Cu(II) remained stiff even after buffer was added and the sample was incubated in contact with the buffer for 24 hr. Vial contains hydrogel sample assembled using F9 4′PyA peptide (1 wt%) and 1 equiv of Cu(II) in buffer (50 mM HEPES, pH 8). Hydrogel sample (300 μL) was set by incubation at 37°C overnight and then the buffer (50 mM HEPES, 5 mM NaCl, pH 8) was added and the picture taken in 24 hr (the vial was inverted for the purposes of the figure, buffer and hydrogel were incubated in the upright position for the experiment).

Table S2. Rheology data of the F9 4′PyA peptide with Cu(II) and Cu(I). Cu(I)Cl was dissolved in 100 mM HCl to prepare a 50 mM stock solution. Hydrogel sample with Cu(I) was prepared by dissolving lyophilised peptide in water and adding Cu(I)Cl stock (75 μL of the mixture with 14 mM F9 4′PyA and 7 mM Cu(I)). Degassed buffer (75 μL of 100 mM HEPES with 35 mM ascorbate, pH 8) was added, the solution was pipette mixed and then rheology measured for 1 hr at 25°C. The rest of the sample was diluted and checked for pH, which was 6.7. We measured evolution of storage modulus for F9 4′PyA with Cu(II) at pH = 6.7. For this, $CuSO_4$ was dissolved in MilliQ water to prepare a 50 mM stock solution. Hydrogel sample with Cu(II) was prepared by dissolving lyophilised peptide in water and adding Cu(II) stock (75 μL of the mixture with 14 mM F9 4′PyA and 7 mM Cu(II)). Then the buffer (75 μL of 100 mM HEPES, pH 6.7) was added and evolution of the storage modulus followed for 1 hr at 25°C. Below G′ values represent values after 1 hr.

Sample	pH	Conc (wt%)	G′ (Pa)
F9 4′PyA with **Cu(II)** (0.5 equiv)	8.0	1	4000
F9 4′PyA with **Cu(II)** (0.5 equiv)	6.7	1	2875
F9 4′PyA with **Cu(I)** (0.5 equiv)	6.7	1	95

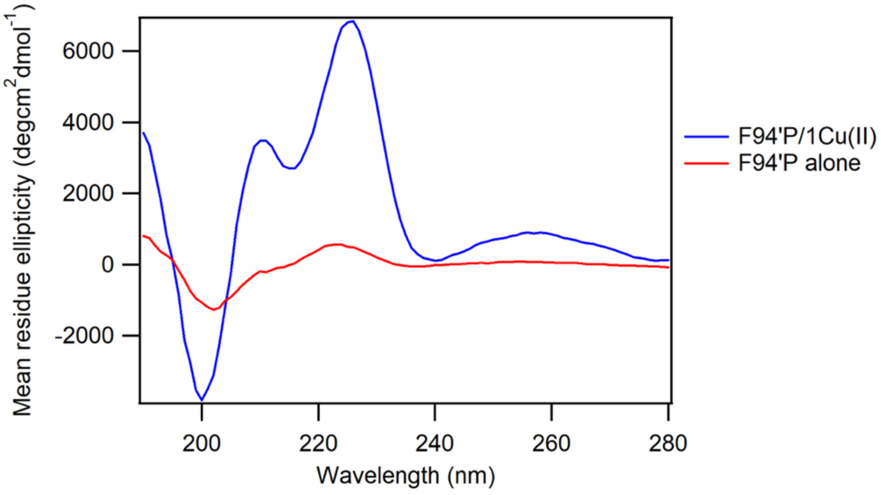

Figure S5. CD spectra of F9 4’PyA peptide with and without Cu(II). The positive signal at 210-230 nm is due to exciton coupling of Phe residues. Samples were prepared by incubating F9 4’PyA peptide (3.5 mM, 0.5 wt%) with 1 equiv of Cu(II) and buffer (50 mM HEPES, pH 8) overnight at 37°C. CD spectra were collected with the pathlength of 0.1 mm and 10 scans for each sample.

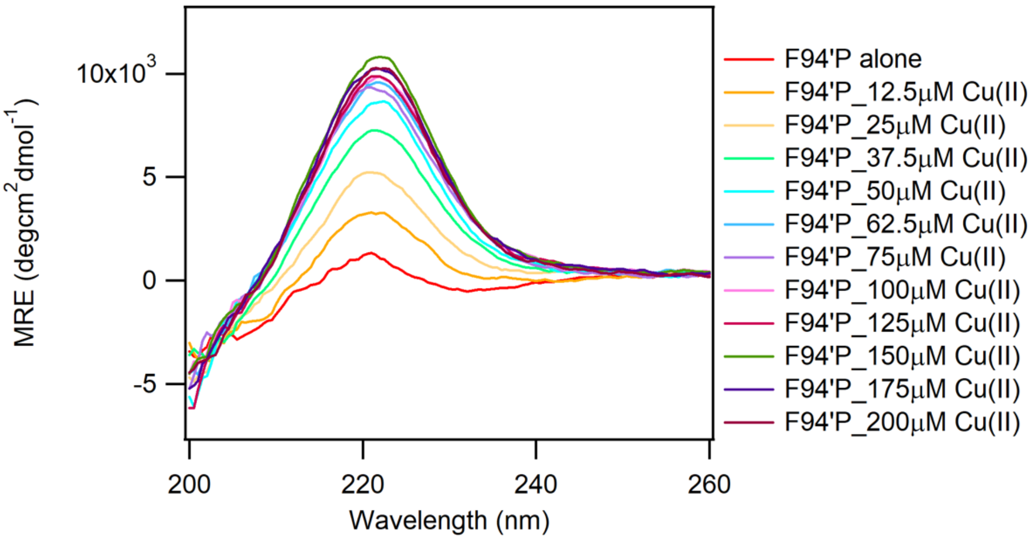

Figure S6. CD spectra showing that the ratio of F9 4’PyA peptide and Cu(II) is 2 to 1. Final concentration of F9 4’PyA peptide is 100 μM; buffer is 5 mM HEPES, pH 8; pathlength of cuvette is 1 mm. Samples were prepared by incubating peptide and Cu(II) in buffer at 37°C for three hours.

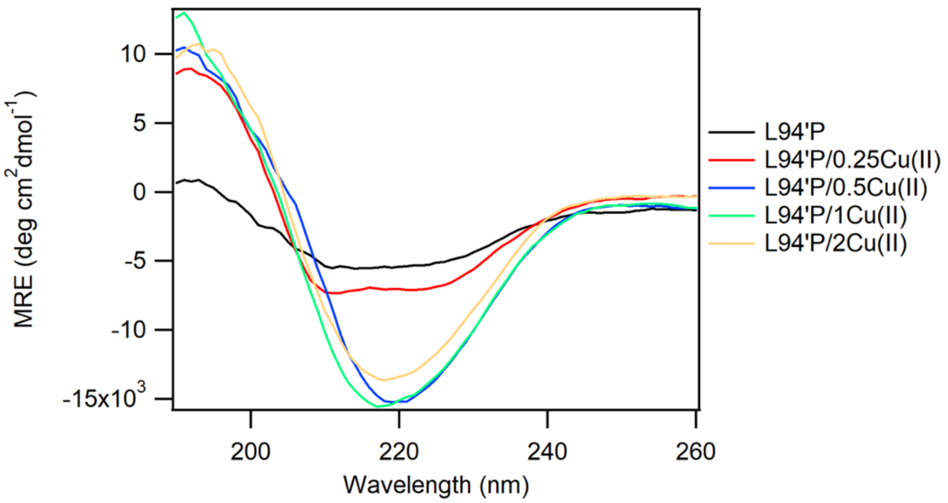

Figure S7. CD spectra of hydrogel samples formed by L9 4′PyA peptide and Cu(II) in buffer (50 mM HEPES, pH 8). Final concentration of peptide is 7.5 mM (1 wt%) and pathlength is 0.1 mm. All samples were incubated overnight at 37°C.

Table S3. Storage modulus G′ in Pa as measured after each cycle of hydrogel sample recovery (after 2 hours). Hydrogel was prepared from 1 wt% of F9 4′PyA peptide with 1 equiv of Cu(II) in buffer (50 mM HEPES, pH 8), subjected to 1000% strain at 6.283 rad/s for 30 s, 25 °C, followed by an oscillation time sweep experiment (0.5% strain) for 2 hours.

Cycles	G′ (Pa)
0	3852
1	3318
2	3320
3	3717
4	3956
5	3326

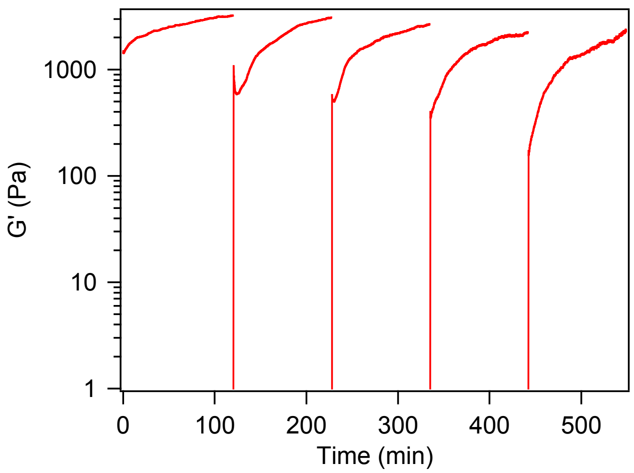

Figure S8. Shear recovery of the hydrogel prepared from **F9 4'PyA** (1 wt%) with Cu(II) (0.5 equiv) in buffer (50 mM HEPES, pH 8). Hydrogel was subjected to 1000% strain at 6.283 rad/s for 30 s, 25 °C, followed by an oscillation time sweep experiment (0.5% strain) for 2 hours to check the sample's recovery after shear.

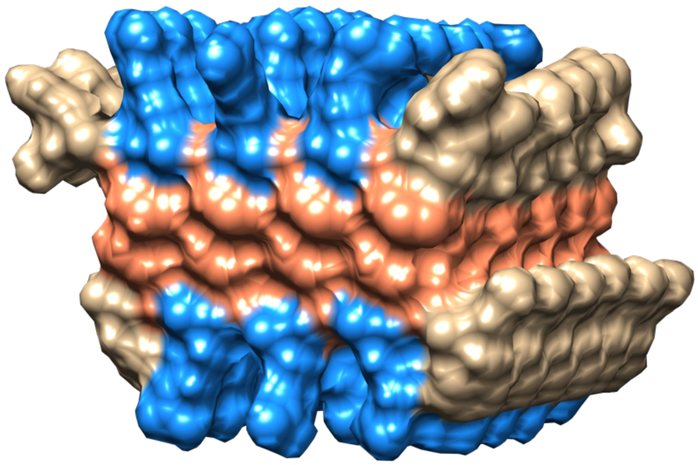

Figure S9. Computational model showing bilayer formed by several F9 4'PyA peptides. Arg side chains are in blue, 4'PyA in light brown and Phe are in orange.

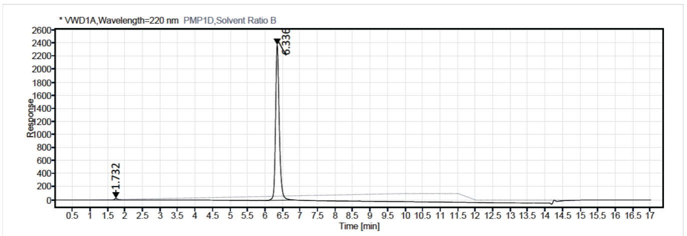

Signal: * VWD1A,Wavelength=220 nm

RT [min]	Width [min]	Area	Height	Area%
1.732	0.09	152.09	25.85	0.86
6.336	0.11	17505.38	2382.50	99.14
	Sum	17657.47		

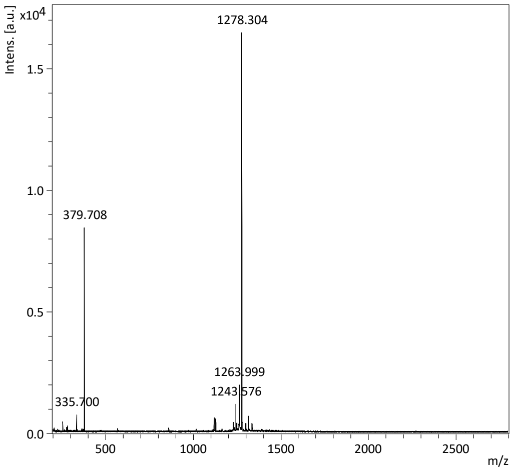

Figure S10. Analytical HPLC chromatogram for purified **L9 3'PyA** peptide (left). Identity of the peptide was confirmed by MALDI-TOF (right). Expected $[M+H]^+$ is 1277.6, observed m/z is 1278.3.

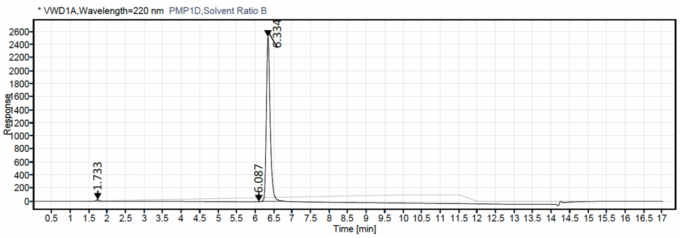

Signal: * VWD1A,Wavelength=220 nm

RT [min]	Width [min]	Area	Height	Area%
1.733	0.08	128.88	25.10	0.71
6.087	0.07	8.38	2.04	0.05
6.334	0.11	18115.44	2524.81	99.25
	Sum	18252.70		

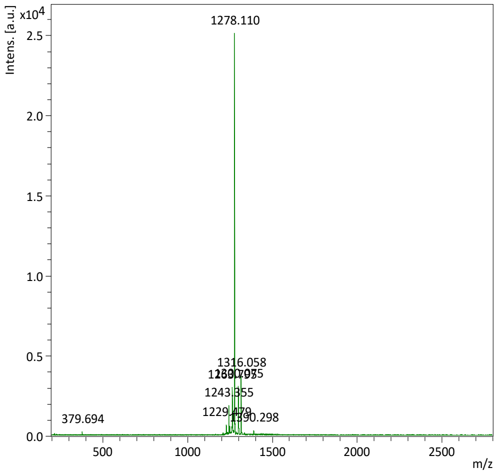

Figure S11. Analytical HPLC chromatogram for purified **L9 4'PyA** peptide (left). Identity of the peptide was confirmed by MALDI-TOF (right). Expected $[M+H]^+$ is 1277.6, observed m/z is 1278.1.

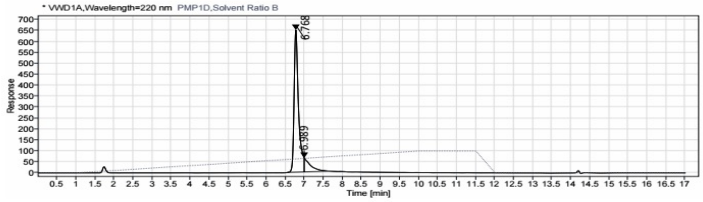

Signal: * VWD1A,Wavelength=220 nm

RT [min]	Width [min]	Area	Height	Area%
6.768	0.12	5274.07	649.30	87.94
6.989	0.19	723.30	63.65	12.06
	Sum	5997.37		

Activate Windows

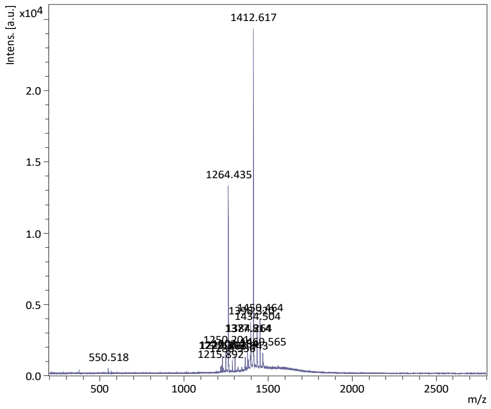

Figure S12. Analytical HPLC chromatogram for purified **F9 2’PyA** peptide (left). Identity of the peptide was confirmed by MALDI-TOF (right). Expected $[M+H]^+$ is 1413.6, observed m/z is 1412.6.

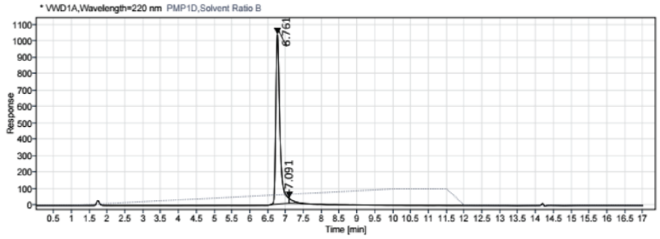

Signal: * VWD1A,Wavelength=220 nm

RT [min]	Width [min]	Area	Height	Area%
6.761	0.12	8116.45	1033.13	97.16
7.091	0.13	237.17	29.97	2.84
	Sum	8353.62		

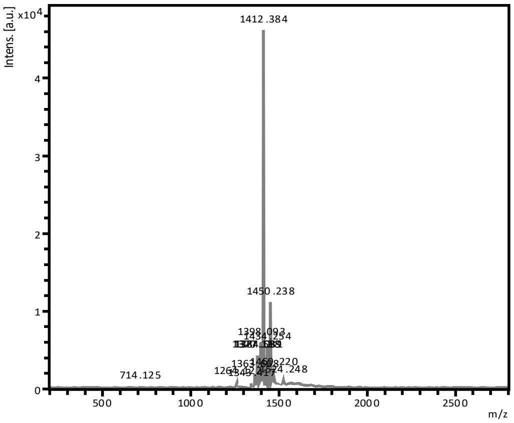

Figure S13. Analytical HPLC chromatogram for purified **F9 3’PyA** peptide (left). Identity of the peptide was confirmed by MALDI-TOF (right). Expected $[M+H]^+$ is 1413.6, observed m/z is 1412.4.

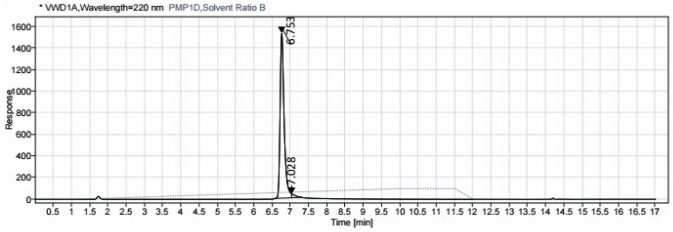

Signal: * VWD1A,Wavelength=220 nm

RT [min]	Width [min]	Area	Height	Area%
6.753	0.11	11612.43	1529.11	98.04
7.028	0.11	232.63	36.22	1.96
	Sum	11845.06		

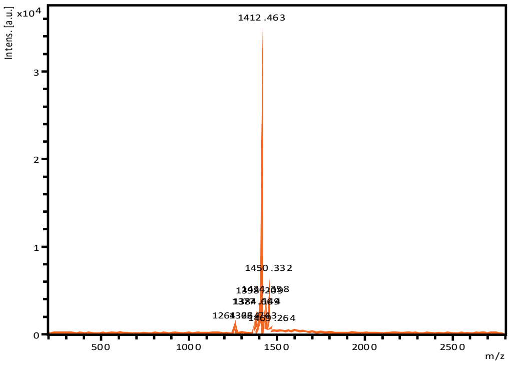

Figure S14. Analytical HPLC chromatogram for purified **F9 4’PyA** peptide (left). Identity of the peptide was confirmed by MALDI-TOF (right). Expected $[M+H]^+$ is 1413.6, observed m/z is 1412.5.

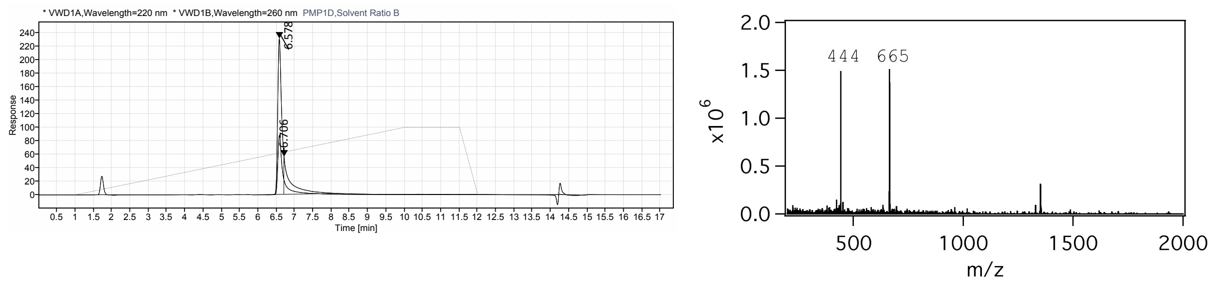

Figure S15. Analytical HPLC chromatogram for purified **FK9 4′PyA** peptide (left). Identity of the peptide was confirmed by ESI-MS (right). Expected for $[M+2H]^{2+}$ is 664.8, for $[M+3H]^{3+}$ is 442.9, observed two peaks at 444 and 665.

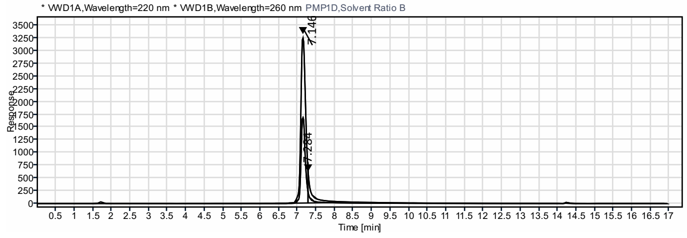

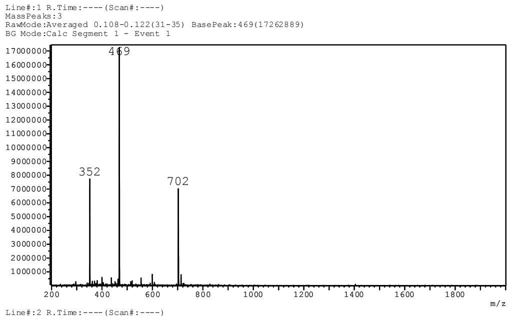

Signal: * VWD1A,Wavelength=220 nm

RT [min]	Width [min]	Area	Height	Area%
7.146	0.14	28970.60	3324.16	86.58
7.284	0.12	4492.28	624.55	13.42
	Sum	33462.88		

Figure S16. Analytical HPLC chromatogram for purified **F9 (2,8) 4′PyA** peptide (left). Identity of the peptide was confirmed by ESI-MS (right). Expected for $[M+2H]^{2+}$ is 700.8, for $[M+3H]^{3+}$ is 470.2, observed peaks at 469 and 702.

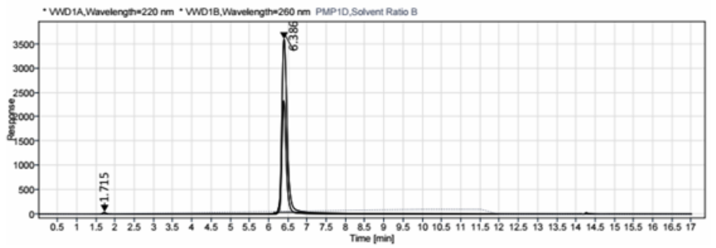

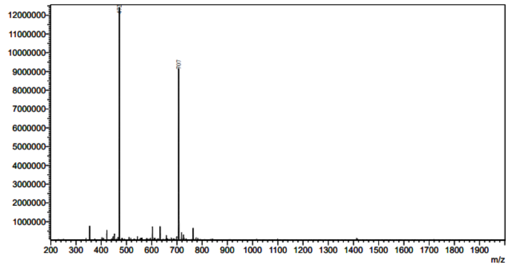

Signal: * VWD1A,Wavelength=220 nm

RT [min]	Width [min]	Area	Height	Area%
1.715	0.08	195.10	48.77	0.58
6.386	0.15	33421.96	3570.44	99.42
	Sum	33617.07		

Figure S17. Analytical HPLC chromatogram for purified **F9 D-4′PyA** peptide (left). Identity of the peptide was confirmed by ESI-MS (right). Expected for $[M+2H]^{2+}$ is 707, for $[M+3H]^{3+}$ is 472, observed two peaks at 472 and 707.

References:

1. Lengyel Z, Rufo CM, Korendovych IV. Preparation and Screening of Catalytic Amyloid Assemblies. Methods Mol Biol. 2018;1777:261-70. doi: 10.1007/978-1-4939-7811-3_16. PubMed PMID: 29744841; PMCID: PMC5951385.
2. Lee M, Wang T, Makhlynets OV, Wu Y, Polizzi NF, Wu H, Gosavi PM, Stöhr J, Korendovych IV, DeGrado WF, Hong M. Zinc-binding structure of a catalytic amyloid from solid-state NMR. Proceedings of the National Academy of Sciences. 2017;114(24):6191-6. doi: doi:10.1073/pnas.1706179114.
3. Land H, Humble MS. YASARA: A Tool to Obtain Structural Guidance in Biocatalytic Investigations. Methods Mol Biol. 2018;1685:43-67. Epub 2017/11/01. doi: 10.1007/978-1-4939-7366-8_4. PubMed PMID: 29086303.
4. Case DA, Cheatham TE, 3rd, Darden T, Gohlke H, Luo R, Merz KM, Jr., Onufriev A, Simmerling C, Wang B, Woods RJ. The Amber biomolecular simulation programs. J Comput Chem. 2005;26(16):1668-88. Epub 2005/10/04. doi: 10.1002/jcc.20290. PubMed PMID: 16200636; PMCID: PMC1989667.
5. Bekker H, Berendsen HJC, Dijkstra EJ, Achterop S, van Drunen R, van der Spoel D, Sijbers A, Keegstra H, Reitsma B, Renardus MKR. Gromacs: A parallel computer for molecular dynamics simulations. Physics computing 92. Singapore: World Scientific; 1993. p. 252-6.
6. Hess B, Bekker H, Berendsen H, Fraaije J. LINCS: A Linear Constraint Solver for molecular simulations. Journal of Computational Chemistry. 1998;18. doi: 10.1002/(SICI)1096-987X(199709)18:123.0.CO;2-H.
7. Darden T, York D, Pedersen L. Particle mesh Ewald: An N·log(N) method for Ewald sums in large systems. The Journal of Chemical Physics. 1993;98(12):10089-92. doi: 10.1063/1.464397.
8. Bussi G, Donadio D, Parrinello M. Canonical sampling through velocity rescaling. J Chem Phys. 2007;126(1):014101. Epub 2007/01/11. doi: 10.1063/1.2408420. PubMed PMID: 17212484.
9. Berendsen HJC, Postma JPM, Gunsteren WFv, DiNola A, Haak JR. Molecular dynamics with coupling to an external bath. The Journal of Chemical Physics. 1984;81(8):3684-90. doi: 10.1063/1.448118.
10. Vreven T, Morokuma K, Farkas O, Schlegel HB, Frisch MJ. Geometry optimization with QM/MM, ONIOM, and other combined methods. I. Microiterations and constraints. J Comput Chem. 2003;24(6):760-9. Epub 2003/04/01. doi: 10.1002/jcc.10156. PubMed PMID: 12666168.
11. Frisch MJ, Trucks GW, Schlegel HB, Scuseria GE, Robb MA, Cheeseman JR, Scalmani G, Barone V, Petersson GA, Nakatsuji H, Li X, Caricato M, Marenich AV, Bloino J, Janesko BG, Gomperts R, Mennucci B, Hratchian HP, Ortiz JV, Izmaylov AF, Sonnenberg JL, Williams, Ding F, Lipparini F, Egidi F, Goings J, Peng B, Petrone A, Henderson T, Ranasinghe D, Zakrzewski VG, Gao J, Rega N, Zheng G, Liang W, Hada M, Ehara M, Toyota K, Fukuda R, Hasegawa J, Ishida M, Nakajima T, Honda Y, Kitao O, Nakai H, Vreven T, Throssell K, Montgomery Jr. JA, Peralta JE, Ogliaro F, Bearpark MJ, Heyd JJ, Brothers EN, Kudin KN, Staroverov VN, Keith TA, Kobayashi R, Normand J, Raghavachari K, Rendell AP, Burant JC, Iyengar SS, Tomasi J, Cossi M, Millam JM, Klene M, Adamo C, Cammi R, Ochterski JW, Martin RL, Morokuma K, Farkas O, Foresman JB, Fox DJ. Gaussian 16 Rev. C.01. Wallingford, CT2016.
12. Becke AD. Density-functional thermochemistry. III. The role of exact exchange. The Journal of Chemical Physics. 1993;98(7):5648-52. doi: 10.1063/1.464913.
13. Lee C, Yang W, Parr RG. Development of the Colle-Salvetti correlation-energy formula into a functional of the electron density. Phys Rev B Condens Matter. 1988;37(2):785-9. Epub 1988/01/15. doi: 10.1103/physrevb.37.785. PubMed PMID: 9944570.
14. Hay PJ, Wadt WR. Ab initio effective core potentials for molecular calculations. Potentials for the transition metal atoms Sc to Hg. The Journal of Chemical Physics. 1985;82(1):270-83. doi: 10.1063/1.448799.
